# The Contribution of Cell Blocks in the Diagnosis of Mediastinal and Hilar Lymphadenopathy Samples From Endobronchial Ultrasound-Guided Transbronchial Needle Aspiration (EBUS-TBNA)

**DOI:** 10.7759/cureus.39673

**Published:** 2023-05-29

**Authors:** Ahmed Aljohaney, Salwa Bakhsh, Manal Khayat

**Affiliations:** 1 Internal Medicine, King Abdulaziz University Hospital, Jeddah, SAU; 2 Pathology, King Abdulaziz University Hospital, Jeddah, SAU; 3 Pathology, King Abdulaziz Medical City Jeddah, Jeddah, SAU

**Keywords:** lymphadenopathy, hilar, mediastinal, cell blocks, ebus-tbna

## Abstract

Background

Endobronchial ultrasound-guided transbronchial needle aspiration (EBUS-TBNA) is a diagnostic procedure that allows clinicians to stage lung cancer by sampling lymph nodes in the mediastinum. EBUS-TBNA is recommended as a first step prior to mediastinoscopy for lung cancer mediastinal staging. This procedure has greatly aided pulmonologists in diagnosing mediastinal pathologies with substantial progress. In this study, our aim is to analyze how cell blocks affect the diagnostic yield of mediastinal and hilar lymphadenopathy using an EBUS cytology needle.

Methods

This retrospective study was conducted at King Abdulaziz University Hospital between May 2021 and September 2021. Patients with mediastinal and hilar lymphadenopathy in the absence of known or suspected primary lung cancer were included. The EBUS procedure was performed using a flexible bronchoscope equipped with a working channel suitable for transbronchial needle aspiration under direct ultrasound guidance. Data were recorded using Microsoft Excel and analyzed using Statistical Package for the Social Sciences (SPSS) v. 26.0 (IBM Corp., Armonk, NY). Diagnostic accuracy measures were determined, and a p-value of 0.05 was established as the final threshold for statistical significance.

Results

The total number of patients in our study was 151. The sensitivity for cytology specimens, histology specimens, and a combined evaluation for the full group of patients was 77.14%, 83.33%, and 87.5%, respectively, with a negative predictive value of 27.22%, 25%, and 21.42%. The diagnostic accuracy for cytology specimens, histology specimens, and a combined evaluation was 71.42%, 76.19%, and 80%, respectively.

Conclusion

Our study found that the combined examination of specimens for both cytology and histology in the diagnosis of lung cancer, sarcoidosis, and tuberculosis resulted in a higher diagnostic yield compared to cytological assessment alone using EBUS-TBNA.

## Introduction

Conventionally, a variety of techniques have been available for mediastinal staging, including mediastinoscopy, blind transbronchial needle, and video-assisted thoracoscopy. However, in 2002, endobronchial ultrasound combined with transbronchial needle aspiration (EBUS-TBNA) was introduced. It uses ultrasound and bronchoscopy to visualize the airway wall and nearby structures and provides real-time guidance for sampling mediastinal and hilar lymph nodes and tumors [1]. EBUS-TBNA was accepted by clinicians in 2007 as a method of choice for mediastinal staging due to its low invasiveness and high sensitivity [1]. Furthermore, EBUS-TBNA has replaced surgical staging as the initial test of choice for evaluating tissue in cases of mediastinal and hilar lymphadenopathy [1-4].

It has been shown that minimally invasive treatments such as EBUS-TBNA can yield high diagnostic value for lung cancer. Additionally, EBUS-TBNA has been utilized in diagnosing metastases from various solid tumors, sarcoidosis, tuberculosis, and lymphoma [2-5]. Recent studies have demonstrated that EBUS-TBNA has a consistent diagnostic yield for most cases with mediastinal and/or hilar lymphadenopathy [2, 6]. Furthermore, the diagnostic yield of EBUS-TBNA for lymphoma has been reported to be within the range of 50-90% [7].

Additional diagnostic tests can be performed by processing EBUS-TBNA material into a cell block preparation (CB). Combining CB with smear preparation has been shown to increase EBUS-TBNA diagnostic yield, and its routine application for the diagnosis of lung cancer has been endorsed by numerous medical associations. However, CB preparations are rarely employed in EBUS-TBNA samples, and little is known about their role in the diagnostic process [8-10].

Most of the initial studies or trials have used highly preselected patient populations, which has resulted in high diagnostic accuracy. While the use of EBUS-TBNA has been well studied in the literature, there is a growing interest in evaluating the diagnostic utility of this modality in unselected patient populations [11], as well as the contribution of CB in the diagnostic process. In this study, we aim to explore how CB affects the accuracy of EBUS cytology needle diagnosis of mediastinal and hilar lymphadenopathy.

## Materials and methods

Population

This retrospective study was conducted at King Abdulaziz University Hospital from May 2021 to September 2021. The study included patients with mediastinal and hilar lymphadenopathy without known or suspected primary lung cancer. Data collection commenced after the hospital institutional review board committee had given its approval.

EBUS-TBNA technique

The EBUS procedure was performed using a flexible bronchoscope (BFUC160F-OL8, Olympus Optical Co Ltd., Tokyo, Japan) equipped with a working channel suitable for TBNA under direct ultrasound guidance and a distal probe that could do linear parallel scans of the mediastinal and peribronchial tissues. Topical lidocaine spray and intravenous midazolam were administered to provide local anesthetic and conscious sedation, respectively, in accordance with established standards. To perform EBUS-TBNA, a special 22-G TBNA needle (NA201SX-4022; Olympus) was used. The internal stylet was used to clean the needle's tip after it had been inserted into the mass. Most passes were carried out using a 20-mL VacLoc syringe (Merit Medical Systems, Inc., South Jordan, UT). The needle was moved from the bronchoscope channel to the tracheal lumen under ultrasound guidance to reach the node or mass, and was then propelled out of the sheath and implanted into the tracheal or bronchial wall. The needle was pushed forward and back while maintaining negative pressure with a syringe at the catheter's proximal end to release the suction before the needle was removed from the target structure.

Lymph node aspirates were smeared onto glass slides, air-dried, and clotted on filter paper. The specimens were then immediately placed in 10% formalin for further laboratory processing to generate cell blocks. No on-site rapid cytology was performed. EBUS-TBNA is considered diagnostic in cases of sarcoidosis or lung cancer if clear and definitive histological and cytological samples of the lymph tissues are obtained. In addition, other diagnoses such as TB were included if found during our investigation. If the procedure failed to provide a definite diagnosis and presented normal lymphoid tissue, it was considered non-diagnostic. All obtained glass slides were examined by a cytology screener and reviewed by two board-certified pathologists.

Statistical analysis

A Microsoft Excel sheet was used to prepare the data sheet to record all data, including demographic characteristics such as name, age, gender, ID, date of procedure, and nationality, as well as indications for performing EBUS, such as lung cancer, sarcoidosis, tuberculosis, and others. The lymph node station was also recorded in the datasheet. The Statistical Package for the Social Sciences (SPSS) v. 26.0 (IBM Corp., Armonk, NY) was used to analyze the data. The results were presented as frequencies and percentages or means ± standard deviation. A Kruskal-Wallis test was used for comparing data in more than two groups, and a Wilcoxon signed-rank test was used for pairwise comparisons. Diagnostic specificity, sensitivity, negative predictive value (NPV), positive predictive value (PPV), and accuracy were determined using accepted definitions. A positive predictive value was defined as the likelihood that a subject with a positive test result will have the disease of interest, whereas a negative predictive value was defined as the likelihood that a subject with a negative test result will not have the disease. Specificity was defined as the probability of receiving a negative test result in a person without the disease, while sensitivity was defined as the probability of receiving a positive test result in a subject with the condition. Diagnostic accuracy was defined as the percentage of subjects that were correctly classified out of all subjects. A p-value of 0.05 was established as the final threshold for statistical significance.

## Results

**Figure 1:**
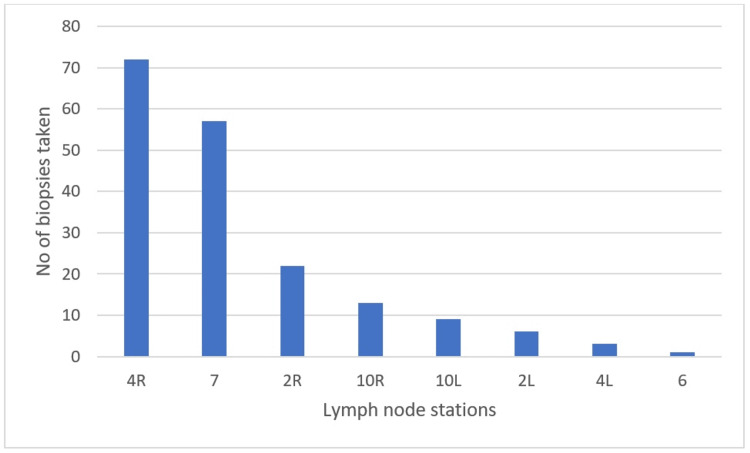
Lymph node stations

Out of the 151 patients who underwent EBUS-TBNA, 101 (66.89%) were male, and the overall mean age of the patients was 52.46 years (range 15-83 years). The EBUS-TBNA evaluation was performed on patients suspected of having lung cancer (n=31, 20.5%), lymphoma (n=16, 10.6%), sarcoidosis (n=24, 15.9%), and tuberculosis (TB) (n=33, 21.9%) (Table 1). The most frequently sampled lymph nodes were the right paratracheal (4R) and subcarinal (7), with a total of 183 lymph nodes sampled (Figure 1).

**Table 1:** Baseline characteristics of patients N: numbers, SD: standard deviation, SOB: shortness of breath, TB: tuberculosis

	No (%)
Gender	
Male	101 (66.89%)
Female	50 (33.11%)
Age (Mean ± SD)	52.46 ± 15.40
Nationality	
Saudi	110 (72.85%)
Non-Saudi	41 (27.15%)
Clinical presentation	
Cough	88 (58.28%)
SOB	57 (37.75%)
Weight loss	49 (32.45%)
Fever	30 (19.87%)
Night sweats	24 (15.89%)
Patient suspected for	
Lung cancer	31 (20.5%)
Lymphoma	16 (10.6%)
Sarcoidosis	24 (15.9%)
TB	33 (21.9%)
Non-definitive diagnosis	47 (31.1%)
Lymph node stations	
4R	72 (47.68%)
7	57 (37.75%)
2R	22 (14.57%)
10 R	13 (8.61%)
10 L	9 (5.96%)
2 L	6 (3.97%)
4 L	3 (1.99%)
6	1 (0.66%)

EBUS-TBNA yielded diagnostic results in 35 cases of cytological smear specimens (23.18%), six cases of histological cell blocks (4.38%), and 89 cases with a combined evaluation (64.96%). The combined evaluation of histology and cytology specimens resulted in the highest rate of positive results and the lowest rate of non-diagnostic results (Table 2).

**Table 2:** Diagnostic and non-diagnostic results of endobronchial ultrasound-guided transbronchial needle aspiration (EBUS-TBNA) for cytology specimens through smears, histology specimens obtained through cell blocks, and a combined evaluation of both CB: cell block

	Cytology	Histology (CB)	Combined Evaluation
Diagnostic	35 (23.18%)	6 (4.38%)	89 (64.96%)
Non-Diagnostic	112 (76.82%)	131 (95.62%)	48 (35.04%)
Total	151	137	137

For the entire group of patients, the sensitivity of specimens for cytology, histology, and a combined evaluation were 77.14%, 83.33%, and 87.5%, respectively, with a negative predictive value (NPV) of 27.22%, 25%, and 21.42%. The diagnostic accuracy was 71.42%, 76.19%, and 80%, respectively (Table 3). Among all the patients who underwent EBUS-TBNA, 46 patients (31.1%) were diagnosed with lung cancer, 24 patients (15.9%) with sarcoidosis, 23 patients (15.2%) with tuberculosis, nine patients (6.6%) with lymphoma, and three patients (2%) with metastatic adenocarcinoma (Table 4).

**Table 3:** Diagnostic parameters of EBUS-TBNA diagnosis PPV: positive predictive value, NPV: negative predictive value, CB: cell block

	Cytology	Histology (CB)	Combined Evaluation
Sensitivity	77.14%	83.33%	87.50%
NPV	27.22%	25%	21.42%
Specificity	100%	100%	100%
PPV	100%	100%	100%
Accuracy	71.42%	76.19%	80%

**Table 4:** Positive EBUS-TBNA results by final diagnosis TB: tuberculosis, CB: cell block

	Cytology	Histology (CB)	Combined evaluation
Lung cancer	6 (12.77%)	--	40 (85.11%)
Lymphoma	3 (30%)	--	6 (60%)
Metastatic adenocarcinoma	--	--	3 (100%)
Sarcoidosis	10 (41.67%)	3 (12.50%)	11 (45.83%)
TB	9 (39.13%)	2 (8.70%)	12 (52.17%)
Others	7 (28%)	1 (4%)	17 (68%)

All positive cytology and histology specimens were found to be accurate in diagnosing with a specificity and positive predictive value of 100%. However, when comparing the sensitivity of cytology specimens with the sensitivity of the combined evaluation, the latter was significantly higher in diagnosing lung cancer (P=<0.001). Similarly, for the diagnoses of sarcoidosis and TB, the sensitivity for the combined evaluation was significantly higher when compared with the cytological and histological evaluations separately (P=<0.001 for each). A significant difference was observed in the sensitivity of diagnosing lung cancer, sarcoidosis, and TB in the cytology evaluation (P=<0.001). The sensitivity of cytology specimens for sarcoidosis was higher when compared with lung cancer and TB. A higher negative predictive value was detected in the cytological evaluation of the sarcoidosis group compared to others. Higher accuracy was found in diagnosing sarcoidosis and TB in their histological evaluation. No cases of lung cancer were diagnosed using histological evaluation, and thus no true negative findings were found. As a result, there was no negative predictive value in the histological evaluation of sarcoidosis and TB. A significant difference was observed in the sensitivity of diagnosing lung cancer, sarcoidosis, and TB in the combined evaluation of both cytological and histological specimens (P=0.032). There was also a significant difference observed in the accuracy of different diagnoses in the combined evaluation of cytology and histology (P=0.041). However, no significant difference was found in the negative predictive value of lung cancer, sarcoidosis, and TB in the combined evaluation (Table 5).

**Table 5:** Sensitivity, negative predictive value (NPV), diagnostic accuracy, specificity, and positive predictive value (PPV) of specimens for cytology, histology, or combined obtained through EBUS-TBNA for lung cancer, sarcoidosis, and TB patients NPV: negative predictive value, PPV: positive predictive value, NS: non-significant, *: P=<0.001;

Results of EBUS-TBNA	Lung Cancer (n=46)	Sarcoidosis (n=24)	Tuberculosis (n=23)	p-value
Cytology				
Sensitivity	12.77%	41.67%	39.13%	<0.001
NPV	79.81%	87.8%	85.87%	NS
Accuracy	84.32%	88.6%	82.65%	NS
Specificity	100%	100%	100%	NS
PPV	100%	100%	100%	NS
Histology				
Sensitivity	--	12.50%	8.70%	NS
NPV	--	0	0	NS
Accuracy		100%	100%	NS
Specificity	--	100%	100%	NS
PPV	--	100%	100%	NS
Cytology and Histology				
Sensitivity	85.11%*	46%*	52.17%*	0.032
NPV	87.24%	66.67%	83.33%	NS
Accuracy	95.11%	70.87%	88.87%	0.041
Specificity	100%	100%	100%	NS
PPV	100%	100%	100%	NS

## Discussion

The results of this study confirmed that adding cell block evaluation to cytological assessment in samples obtained by EBUS-TBNA improves diagnostic sensitivity. The sensitivity of EBUS-TBNA was found to be 77.14% for cytology specimens, 83.33% for histology specimens, and 87.5% for combined evaluation. The negative predictive value for the same specimens was 27.22%, 25%, and 21.42%, respectively. The diagnostic accuracy was found to be 71.42% for cytology specimens, 76.19% for histology specimens, and 80% for combined evaluation. Compared to Žemaitis et al.'s retrospective study, our results show higher sensitivity percentages [11]. Their study, conducted in 2018, was performed on 296 patients between 2009 and 2012. They found that histology specimens had a sensitivity of 70.1%, cytology specimens had a sensitivity of 65.7%, and combined results had a sensitivity of 80.7%. They also reported diagnostic accuracy of 73.5%, 76.4%, and 85.1% for the entire group of patients. Furthermore, their study showed a diagnostic accuracy of 92.1% for lung cancer, with a sensitivity rate of 84.1% [11]. They reported sensitivity and diagnostic accuracies of 78.8% and 94.9%, respectively, for sarcoidosis [11]. However, their reported percentage for diagnostic accuracy was higher than what we found in our study. Another retrospective study of 350 patients conducted from 2008 to 2014 found that EBUS-TBNA had an 89% sensitivity, 86% diagnostic yield, and a 79% negative predictive value, which were all higher than our findings [12]. In 2008, Lee et al. conducted a prospective study using EBUS-TBNA to detect NSCLC. The study aimed to access lymph nodes in 91 patients and revealed a sensitivity rate of 93.8% and a negative predictive value of 96.9%. Their confirmation was based on either suspicion or having a histologically proven NSCLC in lymph nodes with a diameter between 5 and 20 mm [13]. According to a retrospective study of 450 patients conducted between 2008 and 2010, the diagnostic accuracy, sensitivity, and specificity were 93.1%, 100%, and 95.1%, respectively [14].

According to multiple studies, the diagnostic utility of EBUS-TBNA heavily relies on the technique and expertise of the performing bronchoscopist. The representativeness of EBUS-TBNA samples has improved over time as bronchoscopists' skills have enhanced. Learning and maintaining this approach requires significant effort. Another factor that could impact the sensitivity of EBUS-TBNA is the method used to select patients for the procedure. A meta-analysis showed that trials that included patients based on a positive CT or PET scan result had a higher sensitivity (94%, 95%, and CI of 93 to 96%) [15-17].

Our results showed that out of the 151 patients who underwent EBUS-TBNA, 47 were diagnosed with lung cancer (31.1%), 24 with sarcoidosis (15.9%), 23 with tuberculosis (15.2%), ten with lymphoma (6.6%), and three with metastatic adenocarcinoma (2%). A retrospective study conducted in 2012 on 129 patient samples found that lung cancer was detected in 81% of cases, extrapulmonary carcinoma in 10%, sarcoidosis in 4%, lymphoma in 2.7%, and tuberculosis in 0.9% of cases [18]. The study by Madan et al. found that EBUS-TBNA was diagnostic in 80.9%, 84.8%, and 75% of sarcoidosis, tuberculosis, and lung cancer patients, respectively [19]. The sensitivity, specificity, PPV, and NPV of EBUS-TBNA were found to be 81.7%, 100%, 100%, and 22.73%, respectively [11].

Pulmonologists and surgeons are convinced that EBUS-TBNA is superior to standard TBNA and an appropriate alternative to mediastinoscopy in lung cancer patients, and the lung cancer care landscape is changing accordingly. Oncologists are more interested in acquiring tissue to obtain relevant samples for personalized care of patients with advanced lung cancer. EBUS-TBNA has proven to be a reliable method in this regard. Many oncologists acknowledge the advantages of EBUS-TBNA because it allows for the sampling of many tumor metastatic areas, which is crucial as tumor heterogeneity becomes more apparent [19]. EBUS-TBNA can be used to re-characterize tumor phenotypes and genotypes following disease progression since it is easy to repeat. In patients with isolated mediastinal lymphadenopathy, where the differential diagnosis is frequently between sarcoidosis, tuberculosis, and lymphoma, EBUS-TBNA has been proven to be beneficial. EBUS-TBNA is also useful for detecting mycobacterial disease in tuberculosis-endemic regions and is welcomed by microbiologists and infectious disease clinicians because it improves the culture-positive rate [20].

The information obtained from EBUS-TBNA cytology samples may not always be sufficient. Additional tissue may need to be obtained, which can be accomplished by converting the collected material into cell blocks. Immunohistochemical labeling and histological diagnosis can then be performed. New treatments for lung cancer have been introduced with varying degrees of success and toxicity, requiring an accurate classification based on pathology. Therefore, cell blocks can be recommended to aid in the diagnosis of lung cancer as they can contribute up to 47.9% of aspirates obtained through EBUS-TBNA and up to 37.6% for definitive diagnosis. A study has shown that the diagnostic yield of EBUS-TBNA was enhanced from 72.9% to 80% in 26.4% of patients [21, 22]. In addition, being a minimally invasive procedure, EBUS-TBNA has negligible complications as compared to conventional transthoracic fine needle aspiration [18, 23].

The study has several limitations that should be considered when interpreting the results. The sample size was relatively small, which could limit the generalizability of the findings. Furthermore, the results could have been variable based on different laboratory methods and references used. The retrospective design could introduce bias, and the lack of blinding may have affected the accuracy of the analysis. The study was conducted at a single center, which may limit the generalizability of the findings to other healthcare settings. The study did not compare the diagnostic yield of EBUS-TBNA to other diagnostic methods, which could provide more information on the overall accuracy of the procedure. Finally, the experience level of the clinicians performing EBUS-TBNA may affect the diagnostic yield of the procedure.

## Conclusions

For evaluating mediastinal and hilar adenopathy, the EBUS-TBNA test remains the gold standard. The approach has been refined since its inception to eliminate unnecessary steps, reduce procedure time, and increase diagnostic yield. Our study found that the combined evaluation of cytology and histology specimens in the diagnosis of lung cancer, sarcoidosis, and tuberculosis demonstrated that EBUS-TBNA had a higher diagnostic yield.
